# Liquid biopsy for therapy monitoring in early-stage non-small cell lung cancer

**DOI:** 10.1186/s12943-021-01371-1

**Published:** 2021-06-01

**Authors:** Misako Nagasaka, Mohammed Hafiz Uddin, Mohammed Najeeb Al-Hallak, Sarah Rahman, Suresh Balasubramanian, Ammar Sukari, Asfar S. Azmi

**Affiliations:** 1 Department of Oncology, Wayne State University School of Medicine, Karmanos Cancer Institute, 4100 John R, Detroit, MI 48201 USA; 2 Division of Neurology, Department of Internal Medicine, St. Marianna University School of Medicine, Kawasaki, Kanagawa Japan; 3 Department of Cell and Molecular Biology, Grand Valley State University, Allendale, MI 49401 USA

**Keywords:** Liquid biopsy, Circulating nucleic acid, ctDNA, ctRNA, miRNA, lncRNA, Therapy monitoring, minimal residual disease (MRD), Early-stage non-small cell lung cancer (NSCLC)

## Abstract

Liquid biopsy is now considered a valuable diagnostic tool for advanced metastatic non-small cell lung cancer (NSCLC). In NSCLC, circulating tumor DNA (ctDNA) analysis has been shown to increase the chances of identifying the presence of targetable mutations and has been adopted by many clinicians owing to its low risk. Serial monitoring of ctDNA may also help assess the treatment response or for monitoring relapse. As the presence of detectable plasma ctDNA post-surgery likely indicates residual tumor burden, studies have been performed to quantify plasma ctDNA to assess minimal residual disease (MRD) in early-stage resected NSCLC. Most data on utilizing liquid biopsy for monitoring MRD in early-stage NSCLC are from small-scale studies using ctDNA. Here, we review the recent research on liquid biopsy in NSCLC, not limited to ctDNA, and focus on novel methods such as micro RNAs (miRNA) and long non-coding (lncRNA).

## Introduction

Liquid biopsy has been incorporated in clinical practice as a valuable diagnostic tool for advanced metastatic non-small cell lung cancer (NSCLC). In NSCLC, circulating tumor DNA (ctDNA) analysis is known to increase the chances of identifying targetable mutations and has been postulated clinically as a safer alternative than tissue biopsies.

Circulating cell-free DNA (cfDNA) in the peripheral blood originating from tumors, either in association with nucleosome (80–200 bp) or encapsulated in extracellular vesicles, is called ctDNA. ctDNA makes its way into the circulation during apoptosis and necrosis of the tumor cells [1]. The increased level of ctDNA in cancer patients results from limited infiltration of immune cells at the tumor site [2]. Due to intra-tumoral heterogeneity, detection of ctDNAs in liquid biopsies has some advantages over tumor tissue biopsies. ctDNA may offer a representative view of the genetic variations present in the tumor [3] and may provide a “real-time” assessment of the tumor burden, given the typical short half-life of cfDNA (between 16 min and 2.5 h) [4–6].

Typically, the ctDNA fractions in a total cfDNA biofluid samples are very low [7–9], which depends on cancer type and stage, requiring the necessity of highly sensitive detection technologies. Besides quantitative PCR (qPCR) and next-generation sequencing (NGS), recently developed techniques, such as digital PCR (dPCR), droplet digital PCR (ddPCR), and beads emulsion amplification magnetics (BEAMing) are in use to analyze ctDNA [1]. Sequencing approaches include CAncer Personalized Profiling by deep sequencing (CAPP-Seq) [10], Tagged AMplicon deep sequencing (TAM-Seq) [11], Safe-sequencing (Safe-Seq) [12], and Duplex sequencing [13]. The Integrated Digital Error Suppression (iDES) has further improved the sensitivity of CAPP-Seq [14].

The noninvasive nature of obtaining ctDNA is an advantage in serial monitoring. Several studies have suggested the potential utility of ctDNA monitoring to detect the presence of minimal residual disease (MRD) post-resection in early-stage NSCLC [7, 15–18]. While there are emerging data on utilizing liquid biopsy to monitor MRD in early-stage NSCLC, most are small-scale studies using ctDNA. This paper reviews the current research on liquid biopsy in NSCLC, not limited to ctDNA, and focuses on novel methods such as micro RNA (miRNA) and long non-coding (lncRNA).

## Main Text

### Clinical use of ctDNA

#### ctDNA and targeted therapy

The most common scenario, where liquid biopsy utilizing ctDNA would be considered in the clinic, is in the context of newly diagnosed advanced metastatic NSCLC. The utilization of ctDNA has been reported to increase the chances of identifying targetable mutations such as EGFR mutations, ALK fusions, ROS1 fusions, BRAF V600E mutation, ERBB2 (HER2) mutations, RET fusions, MET amplification, and MET exon 14 skipping variants [19, 20].

Another common scenario where ctDNA testing could be utilized is perhaps at the time of disease progression in a patient on a tyrosine kinase inhibitor (TKI) treatment. ctDNA can help to identify the mechanism of resistance in such scenarios, and some secondary molecular alterations may be amenable to yet another TKI [21–25].

Indeed, the International Association for the Study of Lung Cancer (IASLC) suggests that liquid biopsy approaches have significant potential to improve patient care, and immediate implementation in the clinic is justified in several therapeutic settings relevant to NSCLC [26].

As the utility of ctDNA testing to guide targeted molecular therapy has been widely assessed through NSCLC patients with EGFR sensitizing mutations, we will use this as an example to reflect on how these tests were incorporated into clinical practice.

The ENSURE study showed that plasma EGFR mutations were useful in detecting patients who would benefit from erlotinib treatment [27]. Data from this study led to the subsequent US Food and Drug Administration (FDA) approval of the Cobas EGFR Mutation Test v2 (Roche Diagnostics, Indianapolis, IN) as the first liquid biopsy test and a companion diagnostic tool to guide treatment choices regarding EGFR TKIs. However, plasma EGFR mutations were detected only in 76.7% of the patients with EGFR mutations, confirmed from tumor tissues. Therefore, plasma-negative patients are recommended to be retested using tissue samples.

Subsequent data from the AURA phase II extension cohort [28] and AURA2 [29] studies led to the approval of the same method to detect the T790M mutation and determine the eligibility of osimertinib post-1st or-2nd generation EGFR TKIs. These studies reported a testing sensitivity of 61.4% using plasma, again suggesting the necessity of tissue testing when plasma-based testing is negative.

In addition to the Cobas EGFR Mutation Test v2, the Therascreen EGFR RGQ PCR Kit (Qiagen, Valencia, CA) has been approved by the European agency to detect EGFR mutations when tumor tissue is insufficient. The Qiagen method demonstrated reasonable mutation status concordance of 94.3% [sensitivity 65.7%, specificity 99.8%, positive predictive value (PPV) 98.6%, negative predictive value (NPV) 93.8%] among 652 matching tissue/cytology and plasma samples [30].

While osimertinib, a third-generation EGFR TKI, is now considered standard first-line therapy in patients with EGFR-mutated NSCLC [31], clinicians in many parts of the world may be bound by their respective health care system. They may still rely on the use of the first or second-generation EGFR TKIs upfront. Upon progression, it is critical to check for the most common resistance mutation on *EGFR*, T790M. If *EGFR*-T790M is detected through ctDNA testing, such patients would be eligible for the use of osimertinib as the second-line agent [28, 29]. However, if T790M is not detected through ctDNA, in that case, it is suggested that a tissue-based biopsy should be performed due to the possible false-negative results using a plasma-based test and also to rule out other resistance mechanisms, including small cell transformation [32].

Moreover, whether be it the first- or second-line therapy, patients inevitably progress on osimertinib. Acquired resistance, such as *EGFR*-C797S [21] and various additional alterations, including mutations (*BRAF*-V600 and *KRAS*) and amplification (*HER2* and *MET*), have been reported [22, 23]. Detection of such alterations utilizing ctDNA may allow subsequent targeted therapy upon progression on osimertinib [24]. Similarly, plasma genotyping has also been used in NSCLC with other molecular alterations [25].

### ctDNA for monitoring MRD after definitive therapy

Although patients with early-stage NSCLC are treated with curative intent, recurrence remains a challenge. Based on the Lung Adjuvant Cisplatin Evaluation (LACE) meta-analysis, the 5-year-survival of patients treated with adjuvant chemotherapy was only 5.4% with a hazard ratio (HR) of death at 0.89 (95% CI 0.82–0.96, *p* = 0.005) [33]. Consolidation immunotherapy with durvalumab after chemoradiation has shown improved overall survival (OS) and progression-free survival (PFS) in unresectable stage III NSCLC. In exchange, over 30% of patients receiving durvalumab experienced at least one grade 3 or 4 adverse events (AEs) [34]. To truly optimize therapy in the curative setting, it is critical to identify those at high risk of recurrence and provide them with effective treatment.

Previous reports have demonstrated a rapid decline in ctDNA levels post-resection in early-stage lung cancer, which suggests the potential role of ctDNA as a measure of detecting recurrence, possibly before overt disease imaging. By identifying six somatic driver mutations of EGFR, KRAS, TP53, BRAF, PIK3CA, and ERBB2 through targeted sequencing utilizing the SV-CA50-ctDNA panel (San Valley Biotech Inc., Beijing, China), Guo et al. reported the changes in tumor and blood ctDNA levels in 41 NSCLC patients before and after surgery. Overall, 19 plasma samples (19/41; 46.3%) were positive for ctDNA before surgery, and among these 19 samples a total of 24 mutations were found including EGFR (17/24; 70.8%), KRAS (2/24; 8.3%), TP53 (3/24; 12.5%), BRAF (1/24; 4.2%), and PIK3CA (1/24; 4.2%). The average plasma ctDNA mutation frequency prior to surgery was 8.88% (range: 0.1–81.06%), whereas post-surgery it drastically reduced to 0.28% (range: 0.00–3.01%) [15]. A similar study by Chen et al. utilized a 50 gene-panel target sequencing to identify somatic mutations in matched tumor tissue DNA and plasma ctDNA samples. They showed that the average post-operation plasma ctDNA mutation frequency was reduced to 0.28% +/− 0.32%, from 1.21% pre-surgery (*p* < .0001) [16]. Detection of ctDNA post-surgical resection may guide clinicians to consider adjuvant therapy, whereas “non-detection” or reduced detection of ctDNA may be utilized to monitor disease recurrence.

If we know that ctDNA is reduced post-resection, could we then utilize it to monitor disease recurrence? How would we know that we are not detecting random DNA and are monitoring for primary disease recurrence?

Newman et al. developed the CAPP-Seq approach, which combines optimized library preparation methods for low DNA input with multiphase bioinformatics, to design a ‘selector’ composed of biotinylated DNA oligonucleotides that target recurrently mutated regions in cancers. The selector is applied to tumor DNA to identify the patient’s cancer-specific genetic aberrations and then directly to circulating DNA to quantify them over time. The authors give a few examples in NSCLC; a patient with stage IB disease who had surgery and another stage IB patient post-SBRT. In both patients, ctDNA was not detectable after definitive therapy, and both had long-term survival. In contrast, another patient who had chemoradiation for stage IIIB disease had a recurrence following an increase in ctDNA concentration post-therapy, although initial imaging showed a near-complete response [10].

Chaudhuri et al. also reported their use of CAPP-Seq to detect ctDNA. In a study of 40 patients treated with curative intent for stage I–III lung cancer, ctDNA was detectable in the first post-treatment blood sample in 94% of the patients experiencing recurrence [17].

In the TRACERx study, pre and post-surgical plasma ctDNA profiling was performed blinded to relapse status in a sub-group of 24 patients. In this study, multiplex-PCR assay panels were synthesized for each patient, targeting clonal and sub clonal single-nucleotide variants (SNVs) selected to track phylogenetic tumor branches in plasma. The multiplex-PCR NGS platform was validated to have a sensitivity of above 99% for detecting SNVs at frequencies above 0.1%, and the specificity of detecting a single SNV was 99.6%. At least two SNVs were detected in ctDNA from early-stage NSCLCs that had been analyzed in their discovery cohort data [35], suggesting biological sensitivity of a two-SNV threshold for ctDNA detection. Therefore, a threshold of two detected SNVs for calling a sample ctDNA-positive was selected. In this study’s validation cohort, at least two SNVs were seen in 13 out of 14 (93%) patients with confirmed NSCLC relapse before or during clinical relapse. In contrast, only 1 out of 10 (10%) patients, with no clinical evidence of NSCLC relapse, had at least two SNVs detected [18].

CAPP-Seq is an economical and ultrasensitive method for quantifying ctDNA. By providing personalized profiling through deep sequencing, the clinical application of CAPP-Seq to monitor and detect disease recurrence may become a vital surveillance tool in the future.

### ctDNA in treatment monitoring for metastatic NSCLC

ctDNA testing may help monitor the therapeutic response in the metastatic setting, especially when there is a known driver, such as the sensitizing *EGFR* mutation. Several studies have evaluated the quantitative changes in serial ctDNA testing and have found a correlation between tumor response and survival.

One of the earlier studies in this area was reported by Mok et al., where ctDNA level was monitored in *EGFR*-mutated patients treated with gemcitabine followed by erlotinib or placebo. In erlotinib-treated patients, the objective response rate (ORR) of those with undetectable ctDNA at 12 weeks was more remarkable than those with detectable ctDNA levels (82.5% vs. 66.7%). Also, the lack of detectability of ctDNA at 12 weeks was associated with a significant increase in PFS and OS (16.6 vs. 7.8 months and 32.4 vs. 17.7 months, respectively) [36].

Similarly, Taus et al. reported 221 plasma ctDNA correlative samples in response to therapy in 33 *EGFR*-mutated NSCLC patients treated with TKI or chemotherapy. In 13 of 14 evaluable cases (93%), ctDNA decrease predicted radiological response. Contrastingly, in 17 of 19 evaluable cases (89%), ctDNA increase predicted radiological progression. In patients with circulating *EGFR* mutation becoming undetectable during follow-up had a significantly better PFS than those with detectable ctDNA, with a median of 295 vs. 55 days (HR 17.1; *p* < 0.001), respectively [37].

Monitoring the treatment effects of immunotherapy on NSCLC patients may be uniquely challenging due to an exacerbated inflammatory response that may mimic cancer progression (pseudo-progression). Goldberg et al. compared longitudinal changes in ctDNA levels of 28 NSCLC patients receiving immunotherapy. In this study, a ctDNA response correlated with superior PFS (HR 0.29; 95% CI, 0.09–0.89; *p* = 0.03) and OS (HR 0.17; 95% CI, 0.05–0.62; *p* = 0.007) [38]. Interestingly, Lee et al. evaluated the association between ctDNA and pseudo-progression in patients with metastatic melanoma treated with immunotherapy. In this study of 125 patients, the number of ctDNA copies was reduced by more than ten folds within 12 weeks of treatment in those who responded to therapy and also accurately identified all nine patients (7%) who had pseudo-progression based on imaging [39]. Future studies to evaluate the combination of ctDNA and imaging may further advance the field by measuring treatment response with greater accuracy.

#### Tumor mutational burden (TMB) in treatment monitoring for metastatic NSCLC

Another promising biomarker for patients receiving immunotherapy isTMB. Traditionally, TMB is measured using whole-exome sequencing (WES) or an NGS cancer gene panel from the tumor tissue. A high TMB correlates with response to immunotherapy in lung cancer [40, 41]. Gandara et al. reported that high blood TMB (bTMB), demonstrated by the FDA-approved FoundationOne (F1) CDx NGS assay [42], and was associated with increased PFS with atezolizumab over docetaxel in the second-line treatment for NSCLC. In patients with bTMB ≥16, a significant increase in PFS with atezolizumab (versus docetaxel) was shown [43]. The B-F1RST study was one of the first prospective trials to evaluate bTMB as a biomarker to predict the benefit of atezolizumab, used in the first-line setting for metastatic NSCLC. Utilizing the same cut-off values of bTMB ≥16, median PFS was 5.0 vs. 3.5 months, and median OS was 23.9 vs. 13.4 months in the bTMB ≥16 vs. < 16 patients, respectively [44]. Wang et al. recently showed the concordance of bTMB with matching tumor TMB calculated using WES. In their validation cohort, the investigators reported that a bTMB ≥6 was associated with superior PFS [45]. Georgiadis et al. developed a novel liquid biopsy method utilizing a hybrid-capture-based 98-kb pan-cancer gene panel with multifactorial error correction and peak-finding algorithm. This approach identified TMB and microsatellite instability (MSI) from plasma cfDNA with a specificity of > 99% (*N* = 163), and sensitivities of 67% (*N* = 15) and 78% (*N* = 23), respectively [46]. While testing for TMB and MSI using liquid biopsy appears promising for real-time evaluation and global measurement of a tumor, testing methods need further optimization and validation of cut-off values to be clinically meaningful.

### What is circulating non-coding RNA (ncRNA)?

Circulating genomic materials in the bloodstream encompass nucleotide fragments of DNA and RNA molecules [1]. The RNAs that originate from tumor cells are called circulating tumor RNAs (ctRNAs) [47], which includes both coding and non-coding RNAs (ncRNAs). The ncRNAs comprise a large group of RNAs without the translational ability, involving micro RNAs (miRNAs), transfer RNAs (tRNAs), long non-coding RNAs (lncRNAs), circular RNAs (circRNAs), small nuclear RNAs (snRNAs), small nucleolar RNAs (snoRNAs), and piwi interacting RNAs (piRNAs) [48, 49].

#### Potential role of ncRNA utilization in monitoring early-stage NSCLC

The detection of ncRNA is a noninvasive, innovative approach for diagnosis [1]. As potential biomarkers, ncRNAs were assessed for their ability to provide qualitative and quantitative information (e.g., expression levels), detect the mutational pattern in the transcript, and identify cancer-associated fusion transcripts or alternative splice variants [48]. Among ncRNAs, miRNAs, lncRNAs, and recently characterized circRNAs are more suitable as biomarkers [48]. Cutting-edge technologies, such as ddPCR and comprehensive characterization of RNA using RNASeq, dramatically enhance the probability of ncRNAs as biomarkers. These are capable of detecting new transcripts and can precisely quantify ncRNAs with higher sensitivity [48].

It has been shown that crizotinib-associated clinical outcomes correlated with EML4-ALK fusion transcripts in NSCLC [50]. Nilsson et al. demonstrated that ctRNA extracted from platelets can detect EML4-ALK rearrangements with 65% sensitivity and 100% specificity [50]. In an index patient, EML4-ALK fusion transcripts reappeared as ctRNA 2 months before radiographic confirmation of crizotinib resistance. Such detection of fusion RNA transcript in the circulation can help monitor and predict targeted therapies’ outcomes [50].

### miRNAs as biomarkers

In 1993, after the discovery of miRNA in *Caenorhabditis elegans* as an LIN-4 gene transcript [51, 52], the miRNA field advanced immensely with an in-depth understanding of its role in human pathobiology [53]. miRNAs are classified as the main category of small ncRNAs. miRNAs consist of 18–25 nucleotides (nts) [54]. miRNAs represent about 3% of the human genome with 700 members, responsible for the regulation of 20–30% of coding genes [55, 56]. Circulating miRNAs have been investigated as biomarker candidates in various types of cancers [57]. The origin of cancer-associated circulating miRNAs is unclear [58]. They may be secreted by circulating tumor cells, tumor cells from the primary site or by cells that have metastasized. The infiltrating immune cells at the tumor microenvironment may also be considered as a source. However, irrespective of their origin, miRNA can be useful biomarkers for tumor progression [59, 60]. Both radio and chemotherapy can influence the circulating miRNA as evident from several studies [61]. Many patients with early-stage NSCLC require adjuvant chemotherapy. miRNAs may serve as a biomarker to assess such therapy’s sensitivity quite early, which playing an important role in patient management decisions and reducing unnecessary therapeutic toxicities [57]. Table 1 summarizes various ctRNAs assessed as a biomarker in NSCLC.

**Table 1:** ctRNAs as a biomarkers in NSCLC

ctRNAs	Expression	Function	Bio-fluids	Patients types	References (PMID)		
**long non coding RNA (lncRNA)**							
lncRNA MALAT1	Down	Diagnostic	Whole blood	NSCLC	26,137,228; 24,313,945		Patients vs healthy controls
lncRNA NR-026689	Up	Diagnostic	Whole blood	NSCLC	26,908,441		Patients vs healthy controls
lncRNA XIST	Up	Diagnostic	Serum	NSCLC	26,339,353		Patients vs healthy controls
lncRNA HIF1A-AS1	Up	Diagnostic	Serum	NSCLC	26,339,353		Patients vs healthy controls
lncRNA SOX2OT		Diagnostic and prognostic	Serum	NSCLC	29,504,701		Patients vs healthy controls
lncRNA ANRIL		Diagnostic and prognostic	Serum	NSCLC	29,504,701		Patients vs healthy controls
lncRNA16 (ENST00000539303)	Up	Diagnostic	Plasma	NSCLC	27,999,202		Patients vs healthy controls
lncRNA GAS5	Down	Diagnostic	Plasma	NSCLC	27,631,209		Patients vs healthy controls
lncRNA UCA1	Up	Diagnostic	Plasma	NSCLC	26,380,024		Patients vs healthy controls
lncRNA SPRY4-IT1	Up	Diagnostic and prognostic	Plasma	NSCLC	26,453,113		Patients vs healthy controls
lncRNA ANRIL	Up	Diagnostic and prognostic	Plasma	NSCLC	26,453,113		Patients vs healthy controls
lncRNA NEAT1	Up	Diagnostic and prognostic	Plasma	NSCLC	26,453,113		Patients vs healthy controls
lncRNA RP11-397D12.4	Up	Prognostic	Plasma	NSCLC	26,393,913		Patients vs healthy controls
lncRNA AC007403.1	Up	Prognostic	Plasma	NSCLC	26,393,913		Patients vs healthy controls
lncRNA ERICH1-AS1	Up	Prognostic	Plasma	NSCLC	26,393,913		Patients vs healthy controls
lncRNA AFAP1-AS1		Diagnostic	Serum	NSCLC	29,080,690		
lncRNA DLX6-AS1		Diagnostic	Plasma	NSCLC	31,612,030		
lncRNA NR-026689	Up	Diagnostic or prognostic	Cell lines (human) and whole blood (rat)	NSCLC	26,908,441		
lncRNA SCARNA7		Diagnostic or prognostic	Pleural effusions	NSCLC	31,691,525	EGFR-TKI) therapy monitoring	
lncRNA MALAT1		Diagnostic or prognostic	Pleural effusions	NSCLC	31,691,525	EGFR-TKI) therapy monitoring	
lncRNA NONHSAT017369		Diagnostic or prognostic	Pleural effusions	NSCLC	31,691,525	EGFR-TKI) therapy monitoring	
lncRNA HNF1A-AS1	Up			Lung cancer including NSCLC	25,863,539		
lncRNA CRNDE				Lung cancer including NSCLC	28,940,804; 30,554,121; 30,230,527; 31,275,444		
Combined lncRNA GAS5 and lncRNA SOX2OT		Diagnostic and prognostic	Plasma	NSCLC	31,077,615		
lncRNA SOX2OT, ANRIL with tumor markers CEA, CYFRA21-1 and SCCA		Diagnostic	Serum	NSCLC	29,504,701		
**micro RNA (miRNA /miR)**							
miR-21	Up			Lung cancer including NSCLC	18,719,201		
combination of miR-145 and miR-451		Diagnostic	Plasma or serum	Lung cancer including NSCLC	28,060,535; 23,301,032		
let-7	SNP			NSCLC	18,922,928		
miR-142-3p		Diagnostic		NSCLC	23,410,826		
miR-125	Up	Prognostic	Serum	NSCLC	22,983,388; 23,794,259	Cisplatin and pemetrexed-based chemotherapy	
miR-22	Up	Prognostic	Serum	NSCLC	22,983,388; 23,794,259	Cisplatin and pemetrexed-based chemotherapy	
miR-1290		Prognostic	Serum	Lung cancer including NSCLC	27,325,363	Metastasis	
miR-1246		Prognostic	Serum	Lung cancer including NSCLC	27,325,363	Metastasis	
miR-590-5p		Prognostic	plasma	NSCLC	31,520,555		
miR-141		Diagnostic and prognostic	Serum	NSCLC	31,938,373		
miR-18a	Up	Prognostic	plasma	NSCLC	29,266,846		
miR-20a	Up	Prognostic	plasma	NSCLC	29,266,846		
miR-92a	Up	Prognostic	plasma	NSCLC	29,266,846		
miR-21, miR-148, miR-152 and miR-638		Diagnostic		NSCLC	31,236,600		
miR-375		Diagnostic and prognostic	Serum	NSCLC (Indian patients cohort)	32,007,320		
miR-320a		Diagnostic and prognostic	Serum	NSCLC (Indian patients cohort)	32,007,320		
miR-21		Diagnostic or prognostic	Sputum	NSCLC	30,165,346		
miR-143		Diagnostic or prognostic	Sputum	NSCLC	30,165,346		
miR-155		Diagnostic or prognostic	Sputum	NSCLC	30,165,346		
miR-210		Diagnostic or prognostic	Sputum	NSCLC	30,165,346		
miR-372	=	Diagnostic or prognostic	Sputum	NSCLC	30,165,346		
**circular RNA (circRNA)**							
circ-0005962 (up), circ-0003958(up), circ-0086414 (down), circ-0001936 (down)		Prognostic	Plasma	NSCLC	29,588,350		
circFARSA			Plasma	NSCLC	29,722,168		
**Others**							
mRNA CCL5		Prognostic		NSCLC	31,215,484		
mRNA CLU		Prognostic		NSCLC	31,215,484		
mRNA SPARC		Prognostic		NSCLC	31,215,484		
mRNA SRGN		Prognostic		NSCLC	31,215,484		
mRNA with KRAS mutation		Diagnostic and prognostic		Lung cancer including NSCLC	12,459,728		
snoRD33/66/76		Diagnostic	Plasma	NSCLC	20,663,213		
snoRDs-66			Sputum	NSCLC	27,176,474		
snoRDs-78			Sputum	NSCLC	27,176,474		
snRNA U2 (RNU2–1)		Diagnostic		Lung cancer including NSCLC	23,527,303		

The levels of miR-1246 and miR-1290 in serum were associated with the initiation, progression, and metastasis of NSCLC [62]. miRNA levels correlated with the tumor stage and clinical response and increased by multiple folds compared to healthy controls, suggesting the utility of miR-1246 and miR-1290 as clinical biomarkers [62]. Mutational changes in miRNAs may also predict the outcome of malignancies. Single nucleotide polymorphisms (SNPs) at let-7 complementary sites (LCS) are associated with an increased risk of NSCLC. In a study, 74 NSCLC tissue samples were sequenced to detect mutations at let-7 complementary sites (LCS) in the KRAS 3′ untranslated region. Specific LCS6 allelic variant is speculated to be a potential risk factor for NSCLC [63]. Detection of mutated miRNAs in the body fluids of NSCLC patients can be a noninvasive approach to determine the patients at risk.

Hu et al. investigated serum miRNA levels in long-term (mean survival 49.54 months) and short-term (mean survival 9.54 months) survivors of stage I-IIIa NSCLC [64]. After validation of differentially expressed serum miRNAs in 243 patients, the study suggested a panel of miRNAs, involving miR-486, miR-30d, miR-1, and miR-499, that were linked to survival [64].

In an NSCLC study, patients treated with cisplatin and/or other chemotherapeutic regimens had increased circulating miR-125b. Elevated serum level of miR-125b was indeed associated with inferior OS (*P* = 0.0012) [65]. Upregulation of miR-125b has been reported to inhibit apoptosis caused by cisplatin and associated with cisplatin resistance in breast and ovarian cancers [66, 67]. The level of miR-125b, therefore, may help in deciding platinum-based chemotherapy response in NSCLC patients. Another study identified a set of 14 circulating miRNA including miR-134, miR-200b, miR-574, miR-858, and let-7c to predict grade 3+ radiation-induced cardiac toxicity (RICT) in NSCLC [68].

Among other miRNAs, the diagnostic and prognostic potential of serum miR-141 was demonstrated in a prospective cohort study involving 108 NSCLC patients and 54 matched healthy controls [69]. Receiver operating characteristic (ROC) curve analysis showed significant elevation of miR-141 expression in NSCLC patients (*P* < 0.001). Moreover, the expression of miR-141 could distinguish both lymphatic (*P* = 0.015) and distant (*P* = 0.025) metastasis. Subtype analysis revealed an association between miR-141 expression and OS of lung adenocarcinoma but not squamous cell carcinoma [69]. Using multivariate Cox regression analyses, Xu et al. assessed elevated plasma levels of miR-18a, miR-20a, and miR-92a as prognostic biomarkers for NSCLC [70]. In an approach to combine miRNAs for the diagnosis of NSCLC, Abdollahi et al. suggested a miR-panel (miR-21, miR-148, miR-152 and miR-638) of 96.4% sensitivity and 86.67% specificity [71]. Recently, exosomal plasma miRNA of NSCLC patients namely miR-23b-3p, miR-10b-5p and miR-21-5p significantly correlated with poor OS (hazard ratio between 2.12–2.42). Several miRNAs in sputum, including miR-21, miR-143, miR-155, miR-210, and miR-372, have also been reported to detect NSCLC earlier [72, 73].

### Long non-coding RNAs (lncRNAs) as biomarkers

Aside from miRNAs, another encouraging source of ncRNA-based biomarkers is the lncRNA. lncRNAs are composed of 200 nts or more without any open reading frame but contribute to various cellular activities and diseases, including cancer [74]. There are approximately 60,000 ncRNAs in the human genome; > 70% are lncRNAs [74]. Only ~ 20% of lncRNAs are annotated and shown to modulate diverse cellular functions [75]. The regulation of gene expression by lncRNAs occurs through cis and trans mechanisms [76, 77]. Additionally, lncRNAs can regulate mRNA splicing and subcellular localization of proteins [78]. Therefore, it is not surprising that abnormal lncRNA expression contributes to cancer development and progression [79].

Recent research on lncRNA has revealed several potential biomarkers for the detection and prognosis of NSCLC. lncRNAs XIST and HIF1A-AS1 were significantly elevated in serum samples of patients with NSCLC [80]. Notably, these were significantly reduced after surgery. Patients with NSCLC and the control group were strongly separated in the ROC curve analysis (area under the curve (AUC) of XIST and HIF1A-AS1 were 0.834 and 0.876, respectively). The combination of XIST and HIF1A-AS1 significantly improved the AUC value (0.931) as a predictive biomarker for NSCLC [80]. An increase in exosomal (plasma) lncRNA Distal-less homeobox six antisense RNA 1 (DLX6-AS1) levels were also determined as a potentially promising diagnostic biomarker. The study analyzed 72 serum samples of NSCLC patients and 64 healthy donors and observed an association with advanced disease, lymph node metastasis and poor tumor differentiation. The sensitivity and specificity were 77.5 and 85.9% respectively. Notably, the expression of DLX6-AS1 in the serum decreased after surgical resection [81].

Recently, an association between lncRNAs and EGFR mutation was demonstrated. Using the Clariom D Human chip technology, in the plasma samples collected from pleural effusions, three lncRNAs (SCARNA7, MALAT1, and NONHSAT017369) were found to be significantly associated with EGFR mutation and thus could be considered as biomarkers of therapy monitoring [82]. These biomarkers could help predict *EGFR* mutation status and monitor EGFR-TKI efficacy in patients with *EGFR*-positive NSCLC.

It is evident from studies that the combination of biomarkers significantly increases the diagnostic and prognostic sensitivity and specificity. Plasma levels of GAS5 and SOX2OT lncRNAs together showed superior sensitivity (83.8%) and specificity (81.4%) for the diagnosis and prognosis of NSCLC [64]. Circulating GAS5 in combination with other biomarkers can be helpful in monitoring after surgical treatment for NSCLC patients [83]. In a cohort study, serum lncRNA biomarkers SOX2OT and ANRIL, along with tumor markers CEA, CYFRA21-1, and SCCA, demonstrated higher sensitivity (77.1%) and specificity (79.2%) for NSCLC diagnosis [84].

### circRNAs as biomarkers

circRNAs were described first in the early 1970s as a non-functional by-product of RNA splicing [85]. They are cell lineage-specific, enriched in exosomes, and observed in different body fluids [86]. Due to its enhanced stability compared to other ncRNAs, they are more suitable as biomarkers for cancer diagnosis, monitoring development, and progression [87]. circRNAs are a large unique family of ncRNAs that have joined covalent ends. They intricate in the complex regulation of normal processes via interacting with proteins, nucleic acids, and other molecules [88–90]. Recently, circFARSA has been detected and confirmed in the plasma of patients with NSCLC, with high confidence (*p* < 0.001) [91], which warrants future studies. The average plasma half-life of circRNAs is more than 48 h, which is much longer than mRNAs (~ 10 h) [92]. The hidden 3′ ends of circRNAs protect them against nuclease-mediated degradations compared to other ncRNAs [90, 93]. It is assumed that circRNAs overexpressed in lung cancer cells have the potential to be released in the circulation. Such notion has been validated, and indeed a differential expression of circRNAs has been observed in the plasma of early and late-stage NSCLC patients [94]. The study further identified a panel of circRNAs with prognostic significance in NSCLC, which includes two overexpressed (circ-0005962 and circ-0003958) and underexpressed (has-circ-0086414 and has-circ-0001936) circRNAs [94].

snRNA/snoRNAs play essential roles in the early steps of gene expression [95]. Recently, a panel of two snoRNAs, including snoRD-66 and snoRD-78, have been identified in sputum samples of patients with NSCLC. The panel detected NSCLC with 74.6% sensitivity and 83.6% specificity, which increased further to 89% sensitivity and 89% specificity when combined with miRNAs (miR-21, miR-31, and miR-210) [96].

### Potential roles and challenges of ctDNA analysis

While multiple studies have proven the utility of ctDNA MRD measurement in post-therapy NSCLC patients and may allow personalized MRD-based adjuvant treatment options, they are still challenged by many limitations.

#### Concordance between the tissue and liquid

When utilizing ctDNA to detect MRD, it is vital to confirm the concordance rates between plasma samples and the original tumor. A systemic review and meta-analysis of 32 studies with 4527 advanced NSCLC patients reported a pooled sensitivity and specificity of 0.70 and 0.98 respectively for ctDNA EGFR mutation status in plasma samples compared to tumor tissues [97]. The discordance between tumor tissue and plasma DNA likely arises from the limitations of ctDNA detection.

#### Detection limitation

Data from the TRACERx study demonstrated that tumor volume correlates with the mean plasma VAF of clonal SNVs in ctDNA-positive NSCLC. A primary NSCLC tumor volume of 10 cm^3^ predicts a ctDNA plasma VAF of 0.1%. The tumor size may constrain the sensitivity of clonal-SNV ctDNA. It may not also be helpful for the identification of very early disease recurrence or as an initial approach to screen early-stage NSCLC. Lung nodules smaller than computed tomography resolution could release ctDNA with allele frequencies as low as 0.00018%. This would challenge even the most efficient ctDNA technologies, which have reported detection thresholds of 0.00025% under optimal conditions. While ctDNA-based methods would need improvements in sensitivity thresholds for identifying microscopic diseases, there might still be a challenge of missing very minimal residual disease. Practically, the combination of such technologies with clinical correlation would be critical in evaluating patients.

#### False positives

The specificity of ctDNA testing could also be a confronting issue, as not all mutations detected in plasma are related to a cancerous process. For example, BRAF V600E mutation, which is known as the driver mutation in melanoma and NSCLC, is found more frequently in tissue specimens of benign nevi [98]. Indeed, Di Giorgi et al. reported a case where BRAF V600E mutated DNA was detected in both plasma and formalin-fixed tissue specimens from a man with benign nevi [99].

Furthermore, cfDNA primarily originates from hematopoietic cells, and evidence of somatic mutations in these cells are common with aging (10% in healthy adults > 70 years), defined as clonal hematopoiesis of indeterminate potential (CHIP) [100]. The presence of CHIP does not necessarily imply the presence of cancer and can confound ctDNA testing results. Hu et al. reported that most *JAK2* mutations, some *TP53*, and rare *KRAS* mutations detected in cfDNAs were from clonal hematopoiesis and not tumors. They suggested using paired peripheral blood cells genotyping so that such clonal hematopoiesis-derived mutations would not be misdiagnosed as an occult malignancy [101]. Li et al. reported a novel approach using cfDNA and matching white blood cells with a hybrid capture panel, covering 37 lung cancer-related gene sequencing up to 50,000x raw target coverage, filtering somatic mutations attributable to clonal hematopoiesis. Plasma NGS sensitivity for known oncogenic drivers was 75% (*N* = 68/91), and the specificity was 100% (*N* = 19/19) [102].

In contrast, germline mutations can also complicate ctDNA testing as their mere presence does not necessarily imply a cancerous process. However, a high allele fraction (typically between 30 and 70%) is suspicious for a germline variant than a somatic hit [103].

On a different note, caution must be taken when performing liquid biopsy testing in pregnant women. The fact that fetal cfDNA are shed into the maternal bloodstream [104] may obscure the interpretation.

The optimal timing of ctDNA testing also needs to be further evaluated in prospective studies. While Chen et al. suggested utilizing ctDNA detection on the third day after R0 resection as the post-operative baseline value [105], Szpechcinski et al. reported a drastic increase in plasma DNA levels a week after primary tumor resection, likely due to the surgical trauma [106]. In the metastatic setting, peak concentration of ctDNA early after the start of therapy was reported [107], which is also likely related to the rapid release of tumor DNA by treatment-induced tumor cell death. Distinguishing these and actual residual/resistant disease must be examined further.

In addition to the timing, the definition of a “positive” MRD, as well as standardized criteria to define “response” and “progression” or “relapse” during longitudinal monitoring, are critical to minimize variations among studies and provide reliable practices.

#### Challenges and the prospect of ncRNAs as biomarkers

The unstable nature and very short half-life of naked ncRNAs in plasma compared to ctDNA is a challenge for ncRNAs to be considered as biomarkers [108]. The stability of ctRNA is also dependent on the complexes they form. For example, miRNA in microvesicles are resistant to RNase-mediated degradation compared to protein-bound miRNA [109]. It is indeed difficult for an individual ctRNA to qualify as a candidate biomarker because of its overlapping levels in patients and healthy controls, limiting its sensitivity and specificity [110]. However, there are some advantages of ncRNAs over ctDNAs. The ctDNAs levels in the circulation are very low (between 0.01–1% of total cfDNA), whereas, ncRNAs are far more abundant than ctDNAs. Dysregulation of ncRNAs in different biofluids is frequent, even in the earliest stages of cancer [4, 111]. A combination of various ncRNAs and other established circulation nucleic acids should be considered to enhance ncRNAs’ potential as a non-invasive biomarker.

#### Cost

Collaborators of Ontario Health conducted a systematic review. They determined that utilizing liquid biopsy as a triage test before performing a tissue biopsy was more cost-effective in the setting of detecting *EGFR* T790M resistance mutation post 1st or 2nd generation EGFR TKIs, as the estimated cost was $700 for a liquid biopsy vs. $2500 for a tissue biopsy [112]. Furthermore, upfront NGS testing in patients with metastatic NSCLC can have substantial cost reductions compared to single-gene testing for both Centers for Medicare & Medicaid Services and US commercial payers [113]. The cost-effectiveness of molecular testing in early-stage NSCLC needs further evaluation. It is estimated that the cost of a personalized ctDNA profiling per patient for sequencing of a single tumor region, synthesis of a patient-specific assay panel, and profiling of five plasma samples is US $1750 [17].

### Future directions

Detecting and targeting ctDNA, particularly those with sensitizing molecular mutations, such as *EGFR*, would be especially beneficial in NSCLC with the availability of specific targeted inhibitors. In the recently reported ADAURA study, adjuvant osimertinib demonstrated a 79% reduction in the disease recurrence risk or death in resected stage IB-IIIA EGFR-mutated NSCLC patients (DFS HR 0.21, 95% CI 0.16–0.28, *p* < 0.0001) [114]. In addition to erlotinib, the ongoing ALCHEMIST study is evaluating the utility of crizotinib (ALK inhibitor) and nivolumab (PD1 inhibitor) in completely resected NSCLC post standard care adjuvant therapy. Many other studies are assessing the role of chemotherapy with or without additional treatments, such as immunotherapy in the adjuvant setting. Table 2 shows the ongoing studies utilizing ctDNA monitoring in early-stage NSCLC.

**Table 2:** Ongoing studies utilizing ctDNA monitoring in early-stage NSCLC

NCT#	Type	Scope	Cohorts/Arms	Primary outcome measures	Number to be enrolled
NCT04367311	Phase 2	Adjuvant chemo + atezolizumab in resected NSCLC and clearance of ctDNA	Non-squam: cisplatin, pemetrexed, atezo Squam: cis, docetaxel, atezo	Percentage of pts. with undetectable ctDNA post adjuvant treatment	100
NCT04267237	Phase 2	Atezolizumab +/− RO7198457 (personalized cancer vaccine) following adjuvant chemo in ctDNA positive pts. post resection	Atezo, atezo + RO7198457	DFS	80
NCT01629498	Phase 1/2	Intensity modulated photon (IMRT) or proton radiation in stage II-IIIB NSCLC	IMRT or proton radiation therapy	MTD of IMRT and proton therapy	100
NCT03521154	Phase 3	Osimertinib following chemoradiation in stage III NSCLC (LAURA)	Osimertinib, placebo	PFS	200
NCT04385368	Phase 3	Durvalumab with chemo in resected stage II-III NSCLC (MERMAID-1)	Chemotherapy + durvalumab/placebo	DFS in MRD positive analysis set	332
NCT03774758	Observational	Biomarkers for risk stratification in lung cancer	Benign nodule on screening, incidental benign nodule, presumed lung cancer, suspicious nodule, suspicious incidental nodule, post-treatment lung cancer	Sensitivity and specificity of ct-DNA LUNAR assay,	590
NCT03553550	Observational	Role of ctDNA from liquid biopsy in resected lung tumor (LIBERTI)	Completely resected early stage NSCLC	Correlation between ctDNA after surgery with DFS	500
NCT03517332	Observational	ctDNA exposure in peripheral blood	Healthy volunteer, those with stage 0-IV cancer (i.e. lung, colon, pancreatic)	ctDNA exposure in peripheral blood using a novel process, a feasibility study	10,000
NCT04354064	Observational	ctDNA for early treatment response assessment of solid tumors	Healthy volunteer, those with stage I-IV solid tumors (i.e. lung, breast, colon)	Freedom from progression	3362
NCT03838588 (China)	Observational	Tracking molecular evolution for NSCLC (T-MENC)	Monitoring of ctDNA in stage IB, II, IIIA NSCLC post resection	Clonal evolution assessed with liquid biopsy, concordance of ctDNA with PFS and OS	200

While detectable plasma ctDNA post-surgery appears promising to utilize as a biomarker for MRD, the interpretation of a negative ctDNA test could be a challenge; as there are limitations to the detection sensitivity and there always will remain a chance of false-negative testing. To minimize the deleterious consequences of diagnosing a patient with a false-negative ctDNA result as “cancer-free”, further long-term prospective studies utilizing serial plasma ctDNA monitoring post-resection with endpoints in OS would be of benefit.

At this time, the most robust evidence in liquid biopsies is reported from NSCLC studies, but recently, the role of liquid biopsies are being expanded into multiple tumor types.

Although the National Comprehensive Cancer Network (NCCN) guidelines recommend *RAS*, *BRAF*, and MSI or mismatch repair deficiency (dMMR) testing for all patients with metastatic colon cancer [115], a retrospective study showed that only 40% of these patients received guideline-recommended testing [116]. Direct comparison of tumor tissue-based approach vs. plasma-based ctDNA testing has shown concordance in detecting *APC*, *TP53*, *KRAS*, *NRAS*, and *BRAF* mutations in treating naïve and non-anti-EGFR-treated cohorts of colorectal cancer [117]. With limited tissue availability in some patients, utilizing ctDNA for detecting these markers could increase the number of patients being tested and increase the chance of receiving appropriate targeted therapies. Besides, ctDNA could be used to predict resistance to anti-EGFR therapy. Diaz et al. found that 40% of the patients with metastatic colon cancer, who were initially KRAS wild-type, developed detectable *KRAS* mutations in their serum using ctDNA after 5–6 months of anti-EGFR therapy with panitumumab [118]. In stage III of colon cancer, Tie et al. reported a significant difference in the 3-year recurrence-free interval in patients with detectable vs. undetectable levels of ctDNA after surgery (47%vs 76%, HR 3.8; *p* < 0.001) and after completion of chemotherapy (30% vs. 77%, HR 0.75; p < 0.001) [119]. This suggests that post-surgical and post-chemotherapy ctDNA analyses may identify patients with stage III colon cancer with a high risk of recurrence. Prospective randomized trials (NCT04068103, NCT03803553) are underway to answer these critical questions.

In resectable pancreatic cancer, Groot et al. monitored ctDNA in 59 patients using ddpcr to detect the most commonly seen somatic *KRAS* mutations (G12D, G12V, G12R, and Q61H) [120]. ctDNA was detected in about 50% of the patients before resection, and this was a strong predictor for worse PFS and OS. Those who received neoadjuvant chemotherapy were less likely to have detectable preoperative ctDNA (21% vs. 70%; *p* < 0.001). Detecting ctDNA post-resection was associated with worse OS.

Late recurrence may also be detected by ctDNA in specific settings. A secondary analysis with ctDNA was done in hormone receptor-positive HER2-negative operable breast cancer patients from a randomized clinical study. Presence of ctDNA approximately 5 years after the initial diagnosis positively correlated with late disease recurrence [121].

Interestingly, there is data to support the use of ctDNA testing even in patients with cancers of unknown origin (CUP). Kato et al. reported the genomic landscape of ctDNA testing in 442 patients with CUP. Overall, 80% of patients (*N* = 353/442) had detectable ctDNA and 66% (*N* = 290/442) had at least one characterized alteration, and most (*N* = 289/290) had an alteration that would be hypothetically targetable [122].

Combining different ctRNAs is one strategy to enhance the sensitivity and specificity as a biomarker for assessing disease detection and progression with increased precision. Simultaneous detection of two mRNAs and a lncRNA in serum exosomes was shown to have more excellent diagnostic value in colorectal cancer [123]. The lack of consensus on an optimal method [108] needs to be overcome for less variable recovery rates for the processing of samples. Notably, the advent of a lab-on-a-chip microdevice technology may allow rapid processing of samples [124]. The microfluidic system called integrated comprehensive droplet digital detection (IC3D) can detect a very low level of miRNAs in plasma within few hours [125]. With the promising potential as a biomarker in different cancer progression stages, the recently identified ctRNAs warrant further validation studies.

## Conclusions

Liquid biopsy approaches utilizing ctDNA, miRNA, and lncRNA have a significant potential to improve patient care. Further prospective studies are warranted to optimize their use in early-stage NSCLC.

## Data Availability

NA

## Abbreviations

AE: Adverse events
ALK: Anaplastic lymphoma kinase
APC: Adenomatous polyposis coli
AUC: Area under the curve
BEAMing: Beads emulsion amplification magnetics
BRAF: V-raf murine sarcoma viral oncogene homolog B1
CAPP-Seq: Cancer personalized profiling by deep sequencing
CI: Confidence interval
cfDNA: Cell free DNA
circRNA: Circular RNA
ctDNA: Circulating tumor DNA
ctRNA: Circulating tumor RNA
ddPCR: Droplet digital PCR
dMMR: Mismatch repair deficiency
dPCR: Digital PCR
EGFR: Epidermal growth factor receptor
ERBB2: Erythroblastic oncogene B
FDA: Food and drug administration
HER2: Human epidermal growth factor receptor 2
HR: Hazard ratio
IASLC: International association for the study of lung cancer
iDES: Integrated digital error suppression
KRAS: Kirsten rat sarcoma viral oncogene homolog
LCS: Let-7 complementary sites
lncRNA: Long non-coding RNA
MET: Mesenchymal epithelial transition factor
miRNA: Micro RNA
MRD: Minimal residual disease
MSI: Microsatellite instability
ncRNA: Non-coding RNA
NCCN: National comprehensive cancer network
NGS: Next generation sequencing
NRAS: Neuroblastoma ras viral oncogene homolog
NSCLC: Non-small cell lung cancer
ORR: Objective response rate
OS: Overall survival
PIK3CA: Phosphatidylinositol-4,5-bisphosphate 3-kinase catalytic subunit alpha
piRNA: Piwi interacting RNA
PFS: Progression-free survival
qPCR: Quantitative PCR
RET: Rearranged during transfection
ROC: Receiver operating characteristics
Safe-Seq: Safe-sequencing
SNP: Single nucleotide polymophisms
snRNA: Small nuclear RNA
snoRNA: Small nucleolar RNA
TAM-Seq: Tagged amplicon deep sequencing
TKI: Tyrosine kinase inhibitor
TMB: Tumor mutational burden
TP53: Tumor protein 53
tRNA: Transfer RNA
WES: Whole-exome sequencing

## References

[1] De Rubis G, Rajeev Krishnan S, Bebawy M. Liquid biopsies in cancer diagnosis, monitoring, and prognosis. Trends Pharmacol Sci 2019;40(3):172–186. 10.1016/j.tips.2019.01.006. 10.1016/j.tips.2019.01.006 30736982

[2] Pisetsky DS, Fairhurst AM. The origin of extracellular DNA during the clearance of dead and dying cells. Autoimmunity 2007;40(4):281–284. 10.1080/08916930701358826. 10.1080/08916930701358826 17516210

[3] Murtaza M, Dawson SJ, Pogrebniak K, Rueda OM, Provenzano E, Grant J, et al. Multifocal clonal evolution characterized using circulating tumour DNA in a case of metastatic breast cancer. Nat Commun 2015;6:8760. 10.1038/ncomms9760. 10.1038/ncomms9760 PMC4659935 26530965

[4] Diehl F, Schmidt K, Choti MA, Romans K, Goodman S, Li M, Thornton K, Agrawal N, Sokoll L, Szabo SA, Kinzler KW, Vogelstein B, Diaz Jr LA Circulating mutant DNA to assess tumor dynamics. Nat Med 2008;14(9):985–990. 10.1038/nm.1789. 10.1038/nm.1789 PMC2820391 18670422

[5] EWH T, Chan KCA, Leung S (2003). Rapid clearance of plasma Epstein-Barr virus DNA after surgical treatment of nasopharyngeal carcinoma. Clin Cancer Res.

[6] Lo YM, Zhang J, Leung TN, Lau TK, Chang A, Hjelm NM. Rapid clearance of fetal DNA from maternal plasma. Am J Hum Genet 1999;64(1):218–224. 10.1086/302205. 10.1086/302205 PMC1377720 9915961

[7] Cheng ML, Pectasides Ririni, Hanna GJ, Parsons HA, Choudhury AD, Oxnard GR. Circulating tumor DNA in advanced solid tumors: clinica relevance and future directions. CA Cancer J Clin 2021; 71(2):176–190. 10.3322/caac.21650. 10.3322/caac.21650 33165928

[8] Bettegowda C, Sausen M, Leary RJ, et al. Detection of circulating tumor DNA in early- and late-stage human malignancies. Sci Transl Med 2014; 6(224):224ra24. 10.1126/scitranslmed.3007094. 10.1126/scitranslmed.3007094 PMC4017867 24553385

[9] Diehl F, Li M, Dressman D, et al. Detection and quantification of mutations in the plasma of patients with colorectal tumors. Proc Natl Acad Sci U S A 2005; 102(45):16368–16373. 10.1073/pnas.0507904102. 10.1073/pnas.0507904102 PMC1283450 16258065

[10] Newman AM, Bratman SV, To J, Wynne JF, Eclov NC, Modlin LA et al. An ultrasensitive method for quantitating circulating tumor DNA with broad patient coverage. Nat Med 2014;20(5):548–554. 10.1038/nm.3519. 10.1038/nm.3519 PMC4016134 24705333

[11] Forshew T, Murtaza M, Parkinson C, Gale D, Tsui DW, Kaper F, et al. Noninvasive identification and monitoring of cancer mutations by targeted deep sequencing of plasma DNA. Sci Transl Med 2012;4(136):136ra68. 10.1126/scitranslmed.3003726. 10.1126/scitranslmed.3003726 22649089

[12] Kinde I, Wu J, Papadopoulos N, Kinzler KW, Vogelstein B. Detection and quantification of rare mutations with massively parallel sequencing. Proc Natl Acad Sci U S A 2011;108(23):9530–9535. 10.1073/pnas.1105422108. 10.1073/pnas.1105422108 PMC3111315 21586637

[13] Kennedy SR, Schmitt MW, Fox EJ, Kohrn BF, Salk JJ, Ahn EH, et al. Detecting ultralow-frequency mutations by duplex sequencing. Nat Protoc 2014;9(11):2586–2606. 10.1038/nprot.2014.170. 10.1038/nprot.2014.170 PMC4271547 25299156

[14] Newman AM, Lovejoy AF, Klass DM, Kurtz DM, Chabon JJ, Scherer F, et al. Integrated digital error suppression for improved detection of circulating tumor DNA. Nat Biotechnol 2016;34(5):547–555. 10.1038/nbt.3520. 10.1038/nbt.3520 PMC4907374 27018799

[15] Guo N, Lou F, Ma Y, Li J, Yang B, Chen W, et al. Circulating tumor DNA detection in lung cancer patients before and after surgery. Sci Rep. 2016;6:33519. 10.1038/srep33519 PMC5027588 27641744

[16] Chen K, Zhang J, Guan T, Yang F, Lou F, Chen W, et al. Comparison of plasma to tissue DNA mutations in surgical patients with non-small cell lung cancer. J Thorac Cardiovasc Surg 2017;154(3):1123–1131.e2. 10.1016/j.jtcvs.2017.04.073. 10.1016/j.jtcvs.2017.04.073 28625771

[17] Chaudhuri AA, Chabon JJ, Lovejoy AF, Newman AM, Stehr H, Azad TD, Khodadoust MS, Esfahani MS, Liu CL, Zhou L, Scherer F, Kurtz DM, Say C, Carter JN, Merriott DJ, Dudley JC, Binkley MS, Modlin L, Padda SK, Gensheimer MF, West RB, Shrager JB, Neal JW, Wakelee HA, Loo Jr BW, Alizadeh AA, Diehn M Early detection of molecular residual disease in localized lung cancer by circulating tumor DNA profiling. Cancer Discov 2017;7(12):1394–1403. 10.1158/2159-8290.CD-17-0716. 10.1158/2159-8290.CD-17-0716 PMC5895851 28899864

[18] Abbosh C, Birkbak NJ, Wilson GA, Jamal-Hanjani M, Constantin T, Salari R, et al. Phylogenetic ctDNA analysis depicts early-stage lung cancer evolution. Nature 2017;545(7655):446–451. 10.1038/nature22364. 10.1038/nature22364 PMC5812436 28445469

[19] Leighl NB, Page RD, Raymond VM, Daniel DB, Divers SG, Reckamp KL, Villalona-Calero MA, Dix D, Odegaard JI, Lanman RB, Papadimitrakopoulou VA Clinical utility of comprehensive cell-free DNA analysis to identify genomic biomarkers in patients with newly diagnosed metastatic non-small cell lung cancer. Clin Cancer Res 2019;25(15):4691–4700. 10.1158/1078-0432.CCR-19-0624. 10.1158/1078-0432.CCR-19-0624 30988079

[20] Aggarwal C, Thompson JC, Black TA, Katz SI, Fan R, Yee SS, Chien AL, Evans TL, Bauml JM, Alley EW, Ciunci CA, Berman AT, Cohen RB, Lieberman DB, Majmundar KS, Savitch SL, Morrissette JJD, Hwang WT, Elenitoba-Johnson KSJ, Langer CJ, Carpenter EL Clinical implications of plasma-based genotyping with the delivery of personalized therapy in metastatic non-small cell lung cancer. JAMA Oncol 2019;5(2):173–180. 10.1001/jamaoncol.2018.4305. 10.1001/jamaoncol.2018.4305 PMC6396811 30325992

[21] Thress KS, Paweletz CP, Felip E, Cho BC, Stetson D, Dougherty B, Lai Z, Markovets A, Vivancos A, Kuang Y, Ercan D, Matthews SE, Cantarini M, Barrett JC, Jänne PA, Oxnard GR Acquired EGFR C797S mutation mediates resistance to AZD9291 in non-small cell lung cancer harboring EGFR T790M. Nat Med 2015;21(6):560–562. 10.1038/nm.3854. 10.1038/nm.3854 PMC4771182 25939061

[22] Ortiz-Cuaran S, Scheffler M, Plenker D, Dahmen L, Scheel AH, Fernandez-Cuesta L, et al. Heterogeneous mechanisms of primary and acquired resistance to third-generation EGFR inhibitors. Clin Cancer Res 2016;22(19):4837–4847. 10.1158/1078-0432.CCR-15-1915. 10.1158/1078-0432.CCR-15-1915 27252416

[23] Le X, Puri S, Negrao MV, Nilsson MB, Robichaux J, Boyle T, et al. Landscape of EGFR-dependent and –independent resistance mechanisms to osimertinib and continuation therapy beyond progression in EGFR-mutant NSCLC. Clin Cancer Res 2018;24(24):6195–6203. 10.1158/1078-0432.CCR-18-1542. 10.1158/1078-0432.CCR-18-1542 PMC6295279 30228210

[24] Guibert N, Hu Y, Feeney N, Kuang Y, Plagnol V, Jones G, et al. Amplicon-based next-generation sequencing of plasma cell-free DNA for detection of driver and resistance mutations in advanced non-small cell lung cancer. Ann Oncol 2018;29(4):1049–1055. 10.1093/annonc/mdy005. 10.1093/annonc/mdy005 PMC5913609 29325035

[25] Shaw AT, Solomon BJ, Besse B, Bauer TM, Lin CC, Soo RA, et al. ALK resistance mutations and efficacy of lorlatinib in advanced anaplastic lymphoma kinase-positive non-small-cell lung cancer. J Clin Oncol 2019;37(16):1370–1379. 10.1200/JCO.18.02236. 10.1200/JCO.18.02236 PMC6544460 30892989

[26] Rolfo C, Mack PC, Scagliotti GV, Baas P, Barlesi F, Bivona TG, Herbst RS, Mok TS, Peled N, Pirker R, Raez LE, Reck M, Riess JW, Sequist LV, Shepherd FA, Sholl LM, Tan DSW, Wakelee HA, Wistuba II, Wynes MW, Carbone DP, Hirsch FR, Gandara DR Liquid biopsy for advanced non-small cell lung cancer (NSCLC): A statement paper from the IASLC. J Thorac Oncol 2018;13(9):1248–1268. 10.1016/j.jtho.2018.05.030. 10.1016/j.jtho.2018.05.030 29885479

[27] Wu YL, Zhou C, Liam CK, Wu G, Liu X, Zhong Z, Lu S, Cheng Y, Han B, Chen L, Huang C, Qin S, Zhu Y, Pan H, Liang H, Li E, Jiang G, How SH, Fernando MCL, Zhang Y, Xia F, Zuo Y First-line erlotinib versus gemcitabine/cisplatin in patients with advanced EGFR mutation-positive non-small-cell lung cancer: analyses from the phase III, randomized, open-label, ENSURE study. Ann Oncol 2015;26(9):1883–1889. 10.1093/annonc/mdv270. 10.1093/annonc/mdv270 26105600

[28] Yang JC, Ahn MJ, Kim DW, Ramalingam SS, Sequist LV, Su WC, et al. Osimertinib in pretreated T790M-positive advanced non-small-cell lung cancer: AURA study phase II extension component. J Clin Oncol 2017;35(12):1288–1296. 10.1200/JCO.2016.70.3223. 10.1200/JCO.2016.70.3223 28221867

[29] Goss G, Tsai CM, Shepherd FA, Bazhenova L, Lee JS, Chang GC, Crino L, Satouchi M, Chu Q, Hida T, Han JY, Juan O, Dunphy F, Nishio M, Kang JH, Majem M, Mann H, Cantarini M, Ghiorghiu S, Mitsudomi T Osimertinib for pretreated EGFR Thr790Met-positive advanced non-small-cell lung cancer (AURA2): a multicentre, open-label, single-arm, phase 2 study. Lancet Oncol 2016;17(12):1643–1652. 10.1016/S1470-2045(16)30508-3. 10.1016/S1470-2045(16)30508-3 27751847

[30] Douillard JY, Ostoros G, Cobo M, Ciuleanu T, McCormack R, Webster A, Milenkova T First-line gefitinib in Caucasian EGFR mutation-positive NSCLC patients: a phase-IV, open-label, single-arm study. Br J Cancer 2014;110(1):55–62. 10.1038/bjc.2013.721. 10.1038/bjc.2013.721 PMC3887309 24263064

[31] Ramalingam SS, Vansteenkiste J, Planchard D, Cho BC, Gray JE, Ohe Y, et al. Overall survival with osimertinib in untreated, EGFR-mutated advanced NSCLC. N Engl J Med 2020;382(1):41–50. 10.1056/NEJMoa1913662. 10.1056/NEJMoa1913662 31751012

[32] Vendrell JA, Quantin X, Serre I, Solassol J. Combination of tissue and liquid biopsy molecular profiling to detect transformation to small cell lung carcinoma during osimertinib treatment. Ther Adv Med Oncol 2020;12:1758835920974192. 10.1177/1758835920974192. 10.1177/1758835920974192 PMC7750569 33414847

[33] Pignon JP, Tribodet H, Scagliotti GV, Douillard JY, Shepherd FA, Stephens RJ, et al. Lung adjuvant cisplatin evaluation: a pooled analysis by the LACE collaborative group. J Clin Oncol 2008;26(21):3552–3559. 10.1200/JCO.2e007.13.9030. 10.1200/JCO.2007.13.9030 18506026

[34] Antonia SJ, Villegas A, Daniel D, Vicente D, Murakami S, Hui R (2018). Overall survival with durvalumab after chemotherapy in stage III NSCLC. N Engl J Med.

[35] Jamal-Hanjani M, Wilson GA, Horswell S (2016). Detection of ubiquitous and heterogeneous mutations in cell-free DNA from patients with early-stage non-small-cell lung cancer. Ann Oncol.

[36] Mok T, Wu YL, Lee JS, Yu CJ, Sriuranpong V, Sandoval-Tan J, et al. Detection and dynamic changes of EGFR mutations from circulating tumor DNA as a predictor of survival outcomes in NSCLC patients treated with first-line intercalated erlotinib and chemotherapy. Clin Cancer Res 2015;21(14):3196–3203. 10.1158/1078-0432.CCR-14-2594. 10.1158/1078-0432.CCR-14-2594 25829397

[37] Taus Á, Camacho L, Rocha P, Hardy-Werbin M, Pijuan L, Piquer G, et al. Dynamics of EGFR mutation load in plasma for prediction of treatment response and disease progression in patients with EGFR-mutant lung adenocarcinoma. Clin Lung Cancer 2018;19(5):387–394.e2. 10.1016/j.cllc.2018.03.015. 10.1016/j.cllc.2018.03.015 29656868

[38] Goldberg SB, Narayan A, Kole AJ, Decker RH, Teysir J, Carriero NJ, et al. Early assessment of lung cancer immunotherapy response via circulating tumor DNA. Clin Cancer Res 2018;24(8):1872–1880. 10.1158/1078-0432.CCR-17-1341. 10.1158/1078-0432.CCR-17-1341 PMC5899677 29330207

[39] Lee JH, Long GV, Menzies AM, Lo S, Guminski A, Whitbourne K, et al. Association between circulating tumor DNA and pseudoprogression in patients with metastatic melanoma treated with anti-programmed cell death 1 antibodies. JAMA Oncol 2018;4(5):717–721. 10.1001/jamaoncol.2017.5332. 10.1001/jamaoncol.2017.5332 PMC5885201 29423503

[40] Rizvi NA, Hellmann MD, Snyder A, Kvistborg P, Makarov V, Havel JJ, et al. Mutational landscape determines sensitivity to PD-1 blockade in non-small cell lung cancer. Science 2015;348(6230):124–128. 10.1126/science.aaa1348. 10.1126/science.aaa1348 PMC4993154 25765070

[41] Hellmann MD, Ciuleanu TE, Pluzanski A, Lee JS, Otterson GA, Audigier-Valette C, et al. Nivolumab plus ipilimumab in lung cancer with a high tumor mutational burden. N Engl J Med 2018;378(22):2093–2104. 10.1056/NEJMoa1801946. 10.1056/NEJMoa1801946 PMC7193684 29658845

[42] Frampton GM, Fichtenholtz A, Otto GA, Wang K, Downing SR, He J, Schnall-Levin M, White J, Sanford EM, An P, Sun J, Juhn F, Brennan K, Iwanik K, Maillet A, Buell J, White E, Zhao M, Balasubramanian S, Terzic S, Richards T, Banning V, Garcia L, Mahoney K, Zwirko Z, Donahue A, Beltran H, Mosquera JM, Rubin MA, Dogan S, Hedvat CV, Berger MF, Pusztai L, Lechner M, Boshoff C, Jarosz M, Vietz C, Parker A, Miller VA, Ross JS, Curran J, Cronin MT, Stephens PJ, Lipson D, Yelensky R Development and validation of a clinical cancer genomic profiling test based on massively parallel DNA sequencing. Nat Biotechnol 2013*;*31(11):1023–1031. 10.1038/nbt.2696. 10.1038/nbt.2696 PMC5710001 24142049

[43] Gandara DR, Paul SM, Kowanetz M, Schleifman E, Zou W, Li Y, et al. Blood-based tumor mutational burden as a predictor of clinical benefit in non-small-cell lung cancer patients treated with atezolizumab. Nat Med 2018;24(9):1441–1448. 10.1038/s41591-018-0134-3. 10.1038/s41591-018-0134-3 30082870

[44] Socinski M, Velcheti V, Mekhail T, Chae YK, Leal TA, Dowell JE, Tsai ML, Dakhil CS, Stella P, Shen V, Hu S, Paul SM, Shames DS, Schleifman E, Fabrizio DA, Nowicki M, Yun C, Phan S, Kim ES Final efficacy results from B-F1RST, a prospective phase II trial evaluating blood-based tumour mutational burden (bTMB) as a predictive biomarker for atezolizumab (atezo) in 1L non-small cell lung cancer (NSCLC). Ann Oncol 2019;30(Suppl. 5):v919–v920. 10.1093/annonc/mdz394.081.

[45] Wang Z, Duan J, Cai S, Han M, Dong H, Zhao J, et al. Assessment of blood tumor mutational burden as a potential biomarker for immunotherapy in patients with non-small cell lung cancer with use of a next-generation sequencing cancer gene panel. JAMA Oncol 2019;5(5):696–702. 10.1001/jamaoncol.2018.7098. 10.1001/jamaoncol.2018.7098 PMC6512308 30816954

[46] Georgiadis A, Durham JN, Keefer LA, Bartlett BR, Zielonka M, Murphy D, et al. Noninvasive detection of microsatellite instability and high tumor mutation burden in cancer patients treated with PD-1 blockade. Clin Cancer Res 2019;25(23):7024–7034. 10.1158/1078-0432.CCR-19-1372. 10.1158/1078-0432.CCR-19-1372 PMC6892397 31506389

[47] Stroun M, Anker P, Beljanski M, Henri J, Lederrey C, Ojha M (1978). Presence of RNA in the nucleoprotein complex spontaneously released by human lymphocytes and frog auricles in culture. Cancer Res.

[48] Zaporozhchenko IA, Ponomaryova AA, Rykova EY, Laktionov PP. The potential of circulating cell-free RNA as a cancer biomarker: challenges and opportunities. Expert Rev Mol Diagn 2018;18(2):133–145. 10.1080/14737159.2018.1425143. 10.1080/14737159.2018.1425143 29307231

[49] Danielson KM, Rubio R, Abderazzaq F, Das S, Wang YE. High throughput sequencing of extracellular RNA from human plasma. PLoS One 2017;12(1):e0164644. 10.1371/journal.pone.0164644. 10.1371/journal.pone.0164644 PMC5218574 28060806

[50] Nilsson RJA, Karachaliou N, Berenguer J, et al. Rearranged EML4-ALK fusion transcripts sequester in circulating blood platelets and enable blood-based crizotinib response monitoring in non-small-cell lung cancer. Oncotarget 2016;7(1):1066–1075. 10.18632/oncotarget.6279. 10.18632/oncotarget.6279 PMC4808052 26544515

[51] Lee RC, Feinbaum RL, Ambros V. The C. Elegans heterochronic gene lin-4 encodes small RNAs with antisense complementarity to lin-14. Cell 1993;75(5):843–854. 10.1016/0092-8674(93)90529-y. 10.1016/0092-8674(93)90529-y 8252621

[52] Wightman B, Ha I, Ruvkun G. Posttranscriptional regulation of the heterochronic gene lin-14 by lin-4 mediates temporal pattern formation in C. elegans. Cell 1993;75(5):855–862. 10.1016/0092-8674(93)90530-4. 10.1016/0092-8674(93)90530-4 8252622

[53] O’Brien J, Hayder H, Zayed Y, Peng C. Overview of MicroRNA biogenesis, mechanisms of actions, and circulation. Front Endocrinol (Lausanne) 2018;9:402. 10.3389/fendo.2018.00402. 10.3389/fendo.2018.00402 PMC6085463 30123182

[54] Bartel DP. MicroRNAs: target recognition and regulatory functions. Cell 2009;136(2):215–233. 10.1016/j.cell.2009.01.002. 10.1016/j.cell.2009.01.002 PMC3794896 19167326

[55] Carthew RW. Gene regulation by microRNAs. Curr Opin Genet Dev 2006;16(2):203–208. 10.1016/j.gde.2006.02.012. 10.1016/j.gde.2006.02.012 16503132

[56] Duttagupta R, Jiang R, Gollub J, Getts RC, Jones KW. Impact of cellular miRNAs on circulating miRNA biomarker signatures. PLoS One 2011;6(6):e20769. 10.1371/journal.pone.0020769. 10.1371/journal.pone.0020769 PMC3117799 21698099

[57] Umu SU, Langseth H, Bucher-Johannessen C, Fromm B, Keller A, Meese E, et al. A comprehensive profile of circulating RNAs in human serum*.* R.N.A Biol 2018;15(2):242–250. 10.1080/15476286.2017.1403003. 10.1080/15476286.2017.1403003 PMC5798962 29219730

[58] Schwarzenbach H, Nishida N, Calin GA, Pantel K. Clinical relevance of circulating cell-free microRNAs in cancer. Nat Rev Clin Oncol 2014;11(3):145–156. 10.1038/nrclinonc.2014.5. 10.1038/nrclinonc.2014.5 24492836

[59] Pigati L, Yaddanapudi SCS, Iyengar R, Kim DJ, Hearn SA, Danforth D, Hastings ML, Duelli DM Selective release of microRNA species from normal and malignant mammary epithelial cells. PLoS One 2010;5(10):e13515. 10.1371/journal.pone.0013515. 10.1371/journal.pone.0013515 PMC2958125 20976003

[60] Okada H, Kohanbash G, Lotze MT. MicroRNAs in immune regulation--opportunities for cancer immunotherapy. Int J Biochem Cell Biol 2010;42(8):1256–1261. 10.1916/j.biocel.2010.02.002. 10.1016/j.biocel.2010.02.002 PMC2889234 20144731

[61] Cui M, Wang H, Yao X, et al. Circulating MicroRNAs in Cancer: potential and challenge. Front Genet 2019;10:626. 10.3389/fgene.2019.00626. 10.3389/fgene.2019.00626 PMC6656856 31379918

[62] Zhang WC, Chin TM, Yang H, Nga ME, Lunny DP, Lim EK, et al. Tumour-initiating cell-specific miR-1246 and miR-1290 expression converge to promote non-small cell lung cancer progression. Nat Commun 2016;7:11702. 10.1038/ncomms11702, 1. 10.1038/ncomms11702 PMC4919505 27325363

[63] Chin LJ, Ratner E, Leng S, Zhai R, Nallur S, Babar I, et al. A SNP in a let-7 microRNA complementary site in the KRAS 3′ untranslated region increases non-small cell lung cancer risk. Cancer Res 2008;68(20):8535–8540. 10.1158/0008-5472.CAN-08-2129. 10.1158/0008-5472.CAN-08-2129 PMC2672193 18922928

[64] Hu Z, Chen X, Zhao Y, Tian T, Jin G, Shu Y, et al. Serum microRNA signatures identified in a genome-wide serum microRNA expression profiling predict survival of non-small-cell lung cancer. J Clin Oncol 2010;28(10):1721–1726. 10.1200/JCO.2009.24.9342. 10.1200/JCO.2009.24.9342 20194856

[65] Cui E, Li H, Hua F, Wang B, Mao W, Feng XR, Li JY, Wang X Serum microRNA 125b as a diagnostic or prognostic biomarker for advanced NSCLC patients receiving cisplatin-based chemotherapy. Acta Pharmacol Sin 2013;34(2):309–313. 10.1038/aps.2012.125. 10.1038/aps.2012.125 PMC4011618 22983388

[66] Wang H, Tan G, Dong L, et al. Circulating MiR-125b as a marker predicting chemoresistance in breast cancer. PLoS One 2012;7(4):e34210. 10.1371/journal.pone.0034210. 10.1371/journal.pone.0034210 PMC3327688 22523546

[67] Kong F, Sun C, Wang Z, et al. miR-125b confers resistance of ovarian cancer cells to cisplatin by targeting pro-apoptotic Bcl-2 antagonist killer 1. J Huazong Univ Sci Technolog Med Sci 2011;31(4):543. 10.1007/s11596-011-0487-z. 10.1007/s11596-011-0487-z 21823019

[68] Hawkins PG, Sun Y, Dess RT, Jackson WC, Sun G, Bi N, Tewari M, Hayman JA, Kalemkerian GP, Gadgeel SM, Lawrence TS, Haken RKT, Matuszak MM, Kong FM, Schipper MJ, Jolly S Circulating microRNAs as biomarkers of radiation-induced cardiac toxicity in non-small-cell lung cancer. J Cancer Res Clin Oncol 2019;145(6):1635–1643. 10.1007/s00432-019-02903-5. 10.1007/s00432-019-02903-5 PMC6956699 30923943

[69] Zhao Y (2018). The diagnostic and prognostic role of circulating miR-141 expression in non-small-cell lung cancer patients. Int J Clin Exp Pathol.

[70] Xu X, Zhu S, Tao Z, Ye S. High circulating miR-18a, miR-20a, and miR-92a expression correlates with poor prognosis in patients with non-small cell lung cancer. Cancer Med 2018;7(1):21–31. 10.1002/cam4.1238. 10.1002/cam4.1238 PMC5773999 29266846

[71] Abdollahi A, Rahmati S, Ghaderi B, Sigari N, Nikkhoo B, Sharifi K, et al. A combined panel of circulating microRNA as a diagnostic tool for detection of the non-small cell lung cancer. Q J M 2019;112(10):779–785. 10.1093/qjmed/hcz158. 10.1093/qjmed/hcz158 31236600

[72] Roa WH, Kim JO, Razzak R, du H, Guo L, Singh R, Gazala S, Ghosh S, Wong E, Joy AA, Xing JZ, Bedard EL Sputum microRNA profiling: a novel approach for the early detection of non-small cell lung cancer. Clin Invest Med 2012;35(5):E271. 10.25011/cim.v35i5.18700. 10.25011/cim.v35i5.18700 23043708

[73] Xie Y, Todd NW, Liu Z, et al. Altered miRNA expression in sputum for diagnosis of non-small cell lung cancer. Lung Cancer 2010;67(2):170–176. 10.1016/j.lungcan.2009.04.004. 10.1016/j.lungcan.2009.04.004 PMC2846426 19446359

[74] Iyer MK, Niknafs YS, Malik R, Singhal U, Sahu A, Hosono Y, et al. The landscape of long non-coding RNAs in the human transcriptome. Nat Genet 2015;47(3):199–208. 10.1038/ng.3192. 10.1038/ng.3192 PMC4417758 25599403

[75] Peng W, Wang J, Shan B, Peng Z, Dong Y, Shi W, He D, Cheng Y, Zhao W, Zhang C, Li B, Duan C Diagnostic and prognostic potential of circulating long non-coding RNAs in non small cell lung cancer. Cell Physiol Biochem 2018;49(2):816–827. 10.1159/000493043. 10.1159/000493043 30165346

[76] Guttman M, Amit I, Garber M, French C, Lin MF, Feldser D, et al. Chromatin signature reveals over a thousand highly conserved large non-coding RNAs in mammals. Nature 2009;458(7235):223–227. 10.1038/nature07672. 10.1038/nature07672 PMC2754849 19182780

[77] Wapinski O, Chang HY. Long non-coding RNAs and human disease. Trends Cell Biol 2011;21(6):354–361. 10.1016/j.tcb.2011.04.001. 10.1016/j.tcb.2011.04.001 21550244

[78] Wilusz JE, Sunwoo H, Spector DL. Long non-coding RNAs: functional surprises from the RNA world. Genes Dev 2009;23(13):1494–1504. 10.1101/gad.1800909. 10.1101/gad.1800909 PMC3152381 19571179

[79] Akhade VS, Pal D, Kanduri C. Long non-coding RNA: genome organization and mechanism of action. Adv Exp Med Biol 2017;1008:47–74. 10.1007/978-981-10-5203-3_2. 10.1007/978-981-10-5203-3_2 28815536

[80] Tantai J, Hu D, Yang Y, Geng J (2015). Combined identification of long non-coding RNA XIST and HIF1A-AS1 in serum as an effective screening for non-small cell lung cancer. Int J Clin Exp Pathol.

[81] Zhang X, Guo H, Bao Y, Yu H, Xie D, Wang X. Exosomal long non-coding RNA DLX6-AS1 AS a potential diagnostic biomarker for non-small cell lung cancer. Oncol Lett 2019;18(5):5197–5204. 10.3892/ol.2019.10892. 10.3892/ol.2019.10892 PMC6781719 31612030

[82] Lv P, Yang S, Liu W, Qin H, Tang X, Wu F, et al. Circulating plasma lncRNAs as novel markers of EGFR mutation status and monitors of epidermal growth factor receptor-tyrosine kinase inhibitor therapy. Thorac Cancer 2020;11(1):29–40. 10.1111/1759-7714.13216. 10.1111/1759-7714.13216 PMC6938758 31691525

[83] Kamel LM, Atef DM, Mackawy AMH, Shalaby SM, Abdelraheim N. Circulating long non-coding RNA GAS5 and SOX2OT as potential biomarkers for diagnosis and prognosis of non-small cell lung cancer. Biotechnol Appl Biochem 2019;66(4):634–642. 10.1002/bab.1764. 10.1002/bab.1764 31077615

[84] Xie Y, Zhang Y, Du L, Jiang X, Yan S, Duan W, et al. Circulating long non-coding RNA act as potential novel biomarkers for diagnosis and prognosis of non-small cell lung cancer. Mol Oncol 2018;12(5):648–658. 10.1002/1878-0261.12188. 10.1002/1878-0261.12188 PMC5928376 29504701

[85] Hsu MT, Coca-Prados M. Electron microscopic evidence for the circular form of RNA in the cytoplasm of eukaryotic cells. Nature 1979;280(5720):339–340. 10.1038/280339a0. 10.1038/280339a0 460409

[86] Wang S, Zhang K, Tan S, et al. Circular RNAs in body fluids as cancer biomarkers: the new fontier of liquid biopsies. Mol Cancer 2021;20(1):13. 10.1186/s12943-020-01298-z. 10.1186/s12943-020-01298-z PMC7798340 33430880

[87] Tucker D, Zheng W, Zhang D, Dong X. Circular RNA and its potential as prostate cancer biomarkers. World J Clin Oncol 2020;11(8):563–572. 10.5306/wjco.v11.i8.563. 10.5306/wjco.v11.i8.563 PMC7443832 32879844

[88] Du WW, Zhang C, Yang W, Yong T, Awan FM, Yang BB. Identifying and characterizing circRNA-protein interaction. Theranostics 2017;7(17):4183–4191. 10.7150/thno.21299. 10.7150/thno.21299 PMC5695005 29158818

[89] Rong D, Sun H, Li Z, Liu S, Dong C, Fu K, Tang W, Cao H An emerging function of circRNA-miRNAs-mRNA axis in human diseases. Oncotarget 2017;8(42):73271–73281. 10.18632/oncotarget.19154. 10.18632/oncotarget.19154 PMC5641211 29069868

[90] Azmi AS. Nuclear export mechanisms of circular RNAs: size does matter. Non-coding RNA *Investig* 2018;2. 10.21037/ncri.2018.08.03. 10.21037/ncri.2018.08.03 PMC6214482 30393780

[91] Hang D, Zhou J, Qin N, Zhou W, Ma H, Jin G, Hu Z, Dai J, Shen H A novel plasma circular RNA circFARSA is a potential biomarker for non-small cell lung cancer. Cancer Med 2018;7(6):2783–2791. 10.1002/cam4.1514. 10.1002/cam4.1514 PMC6010816 29722168

[92] Jeck WR, Sharpless NE. Detecting and characterizing circular RNAs. Nat Biotechnol 2014;32(5):453–461. 10.1038/nbt.2890. 10.1038/nbt.2890 PMC4121655 24811520

[93] Meng S, Zhou H, Feng Z, Xu Z, Tang Y, Li P, Wu M CircRNA: functions and properties of a novel potential biomarker for cancer. Mol Cancer 2017;16(1):94. 10.1186/s12943-017-0663-2. 10.1186/s12943-017-0663-2 PMC5440908 28535767

[94] Qiu M, Xia W, Chen R, et al. The circular RNA circPRKCI promotes tumor growth in lung adenocarcinoma. Cancer Res 2018;78(11):2839–2851. 10.1158/0008-5472.CAN-17-2808. 10.1158/0008-5472.CAN-17-2808 29588350

[95] Liao J, Yu L, Mei Y, Guarnera M, Shen J, Li R, Liu Z, Jiang F Small nucleolar RNA signatures as biomarkers for non-small-cell lung cancer. Mol Cancer 2010;9:198. 10.1186/1476-4598-9-198, 1. 10.1186/1476-4598-9-198 PMC2919450 20663213

[96] Su Y, Guarnera MA, Fang H, Jiang F. Small non-coding RNA biomarkers in sputum for lung cancer diagnosis. Mol Cancer 2016;15(1):36. 10.1186/s12943-016-0520-8. 10.1186/s12943-016-0520-8 PMC4866414 27176474

[97] Zhou S, Huang R, Cao Y. Detection of epidermal growth factor receptor mutations in peripheral blood circulating tumor DNA in patients with advanced non-small cell lung cancer: A PRISMA-compliant meta-analysis and systematic review. Medicine (Baltimore) 2020; 99(40):e21965. 10.1097/MD.0000000000021965. 10.1097/MD.0000000000021965 PMC7535563 33019389

[98] Pollock PM, Harper UL, Hansen KS, Yudt LM, Stark M, Robbins CM, Moses TY, Hostetter G, Wagner U, Kakareka J, Salem G, Pohida T, Heenan P, Duray P, Kallioniemi O, Hayward NK, Trent JM, Meltzer PS High frequency of BRAF mutations in nevi. Nat Genet 2003;33(1):19–20. 10.1038/ng1054. 10.1038/ng1054 12447372

[99] De Giorgi V, Pinzani P, Salvianti F, et al. Circulating benign nevus cells detected by ISET techniques: waning for melanoma molecular diagnosis. Arch Dermatol 2010;146(10):1120–1124. 10.1001/archdermatol.2010.264. 10.1001/archdermatol.2010.264 20956643

[100] Jaiswal S, Fontanillas P, Flannick J, Manning A, Grauman PV, Mar BG, Lindsley RC, Mermel CH, Burtt N, Chavez A, Higgins JM, Moltchanov V, Kuo FC, Kluk MJ, Henderson B, Kinnunen L, Koistinen HA, Ladenvall C, Getz G, Correa A, Banahan BF, Gabriel S, Kathiresan S, Stringham HM, McCarthy MI, Boehnke M, Tuomilehto J, Haiman C, Groop L, Atzmon G, Wilson JG, Neuberg D, Altshuler D, Ebert BL Age-related clonal hematopoiesis associated with adverse outcomes. N Engl J Med 2014;371(26):2488–2498. 10.1056/NEJMoa1408617. 10.1056/NEJMoa1408617 PMC4306669 25426837

[101] Hu Y, Ulrich BC, Supplee J, Kuang Y, Lizotte PH, Feeney NB, Guibert NM, Awad MM, Wong KK, Jänne PA, Paweletz CP, Oxnard GR False-positive plasma genotyping due to clonal hematopoiesis. Clin Cancer Res 2018;24(18):4437–4443. 10.1158/1078-0432.CCR-18-0143. 10.1158/1078-0432.CCR-18-0143 29567812

[102] Li BT, Janku F, Jung B, Hou C, Madwani K, Alden R, et al. Ultra-deep next-generation sequencing of plasma cell-free DNA in patients with advanced lung cancers: results from the actionable genome consortium. Ann Oncol 2019;30(4):597–603. 10.1093/annonc/mdz046. 10.1093/annonc/mdz046 PMC6503621 30891595

[103] Singh AP, Cheng H, Guo X, Levy B, Halmos B. Circulating tumor DNA in non–small-cell lung cancer: a primer for the clinician. *J.C.O.* Precis Oncol 2017;1:1–13. 10.1200/PO.17.00054. 10.1200/PO.17.00054 35172511

[104] Bianchi DW, Chiu RWK. Sequencing of circulating cell-free DNA during pregnancy. N Engl J Med 2018;379(5):464–473. 10.1056/NEJMra1705345. 10.1056/NEJMra1705345 PMC10123508 30067923

[105] Chen K, Zhao H, Shi Y, Yang F, Wang LT, Kang G, et al. Perioperative dynamic changes in circulating tumor DNA in patients with lung cancer (DYNAMIC). Clin Cancer Res 2019;25(23):7058–7067. 10.1158/1078-0432.CCR-19-1213. 10.1158/1078-0432.CCR-19-1213 31439586

[106] Szpechcinski A, Chorostowska-Wynimko J, Kupis W, Maszkowska-Kopij K, Dancewicz M, Kowalewski J, Orlowski T Quantitative analysis of free-circulating DNA in plasma of patients with resectable NSCLC. Expert Opin Biol Ther 2012;12(Suppl. 1):S3–S9. 10.1517/14712598.2012.668519. 10.1517/14712598.2012.668519 22559166

[107] Vallée A, Audigier-Valette C, Herbreteau G, Merrien J, Tessonnier L, Théoleyre S, et al. Rapid clearance of circulating tumor DNA during treatment with AZD9291 of a lung cancer patient presenting the resistance EGFR T790M mutation. Lung Cancer 2016;91:73–74. 10.1016/j.lungcan.2015.11.008. 10.1016/j.lungcan.2015.11.008 26612314

[108] Sourvinou IS, Markou A, Lianidou ES. Quantification of circulating miRNAs in plasma: effect of preanalytical and analytical parameters on their isolation and stability. J Mol Diagn 2013;15(6):827–834. 10.1016/j.jmoldx.2013.07.005. 10.1016/j.jmoldx.2013.07.005 23988620

[109] Winther TN, Bang-Berthelsen CH, Heiberg IL, Pociot F, Hogh B (2013). Differential plasma microRNA profiles in HBeAg positive and HBeAg negative children with chronic hepatitis B. PLoS One.

[110] Wang H, Peng R, Wang J, Qin Z, Xue L (2018). Circulating microRNAs as potential cancer biomarkers: the advantage and disadvantage. Clin Epigenetics.

[111] Shigeyasu K, Toden S, Zumwalt TJ, Okugawa Y, Goel A. Emerging role of microRNAs as liquid biopsy biomarkers in gastrointestinal cancers. Clin Cancer Res 2017;23(10):2391–2399. 10.1158/1078-0432.CCR-16-1676. 10.1158/1078-0432.CCR-16-1676 PMC5433899 28143873

[112] Ontario Health (Quality). Lambrinos A, Falk L, Gajic-Veljanoski O, et al. (2020). Cell-free circulating tumour DNA blood testing to detect *EGFR* T790M mutation in people with advanced non-small cell lung Cancer: A Health technology assessment. Ont Health Technol Assess Ser.

[113] Pennell NA, Mutebi A, Zhou ZY, Ricculli ML, Tang W, Wang H (2019). Economic impact of next-generation sequencing versus single-gene testing to detect genomic alterations in metastatic non-small-cell lung cancer using a decision analytic model. J.C.O. Precis Oncol.

[114] Wu YL, Tsuboi M, He J, John T, Grohe C, Majem M, Goldman JW, Laktionov K, Kim SW, Kato T, Vu HV, Lu S, Lee KY, Akewanlop C, Yu CJ, de Marinis F, Bonanno L, Domine M, Shepherd FA, Zeng L, Hodge R, Atasoy A, Rukazenkov Y, Herbst RS Osimertinib in resected EGFR-mutated non-small-cell lung Cancer. N Engl J Med. 2020;383(18):1711–23. 10.1056/NEJMoa2027071. 10.1056/NEJMoa2027071 32955177

[115] National Comprehensive Cancer Network. Colon Cancer (Version 4.2020). https://www.nccn.org/professionals/physician_gls/pdf/colon.pdf. Accessed 21 Mar 2021.

[116] Gutierrez ME, Price KS, Lanman RB, Nagy RJ, Shah I, Mathura S (2019). Genomic profiling for KRAS, NRAS, BRAF, microsatellite instability, and mismatch repair deficiency among patients with metastatic colon cancer. J.C.O. Precis Oncol.

[117] Gupta R, Othman T, Chen C, Sandhu J, Ouyang C, Fakih M. Guardant360 circulating tumor DNA assay is concordant with FoundationOne next-generation sequencing in detecting actionable driver mutations in anti-EGFR naïve metastatic colorectal cancer. Oncologist 2020;25(3):235–243. 10.1634/theoncologist.2019-0441. 10.1634/theoncologist.2019-0441 PMC7066697 32162812

[118] Diaz LA Jr., Williams RT, Wu J, Kinde I, Hecht JR, Berlin J, et al. The molecular evolution of acquired resistance to targeted EGFR blockade in colorectal cancers. Nature 2012;486(7404):537–540. 10.1038/nature11219. 10.1038/nature11219 PMC3436069 22722843

[119] Tie J, Cohen JD, Wang Y, Christie M, Simons K, Lee M, et al. Circulating tumor DNA analyses as markers of recurrence risk and benefit of adjuvant therapy for stage III colon cancer. JAMA Oncol 2019;5(12):1710–1717. 10.1001/jamaoncol.2019.3616. 10.1001/jamaoncol.2019.3616 PMC6802034 31621801

[120] Groot VP, Mosier S, Javed AA, Teinor JA, Gemenetzis G, Ding D, et al. Circulating tumor DNA as a clinical test in resected pancreatic cancer. Clin Cancer Res 2019;25(16):4973–4984. 10.1158/1078-0432.CCR-19-0197. 10.1158/1078-0432.CCR-19-0197 PMC7403524 31142500

[121] Sparano J, O’Neill A, Alpaugh K, Wolff AC, Northfelt DW, Dang CT, Sledge GW, Miller KD Association of circulating tumor cells with late recurrence of estrogen receptor-positive breast cancer: a secondary analysis of a randomized clinical trial. JAMA Oncol 2018;4(12):1700–1706. 10.1001/jamaoncol.2018.2574. 10.1001/jamaoncol.2018.2574 PMC6385891 30054636

[122] Kato S, Krishnamurthy N, Banks KC, De P, Williams K, Williams C, et al. Utility of genomic analysis in circulating tumor DNA from patients with carcinoma of unknown primary. Cancer Res 2017;77(16):4238–4246. 10.1158/0008-5472.CAN-17-0628. 10.1158/0008-5472.CAN-17-0628 PMC5729906 28642281

[123] Dong L, Lin W, Qi P, et al. Circulating Long RNAs in serum extracellular vesicles: their characterization and potential application as biomarkers for diagnosis of colorectal Cancer. Cancer Epidemiol Biomark Prev 2016;25(7):1158–1166. 10.1158/1055-9965.EPI-16-0006. 10.1158/1055-9965.EPI-16-0006 27197301

[124] Potrich C, Vaghi V, Lunelli L, et al. OncomiR detection in circulating body fluids: a PDMS microdevice perspective. Lab Chip 2014;14(20):4067–4075. 10.1039/c4lc00630e. 10.1039/c4lc00630e 25178053

[125] Zhang K, Kang D, Ali MM, et al. Digital quantification of miRNA directly in plasma using integrated comprehensive droplet digital detection. Lab Chip 2015;15(21):4217–4226. 10.1039/c5lc00650c. 10.1039/c5lc00650c PMC4631652 26387763

